# Brolucizumab-Associated Ocular Complications with Concomitant Sub-Tenon Triamcinolone Acetonide: A Multicenter Retrospective Study

**DOI:** 10.3390/jcm15218507

**Published:** 2026-11-02

**Authors:** Ryo Ando, Aki Kato, Kenichi Kimoto, Maho Itotani, Taiichi Hikichi, Goro Watanabe, Taku Sato, Hideho Matsuda, Satomi Shiose, Hisashi Matsubara, Susumu Ishida

**Affiliations:** 1 Department of Ophthalmology, Faculty of Medicine, Graduate School of Medicine, Hokkaido University, Sapporo 060-8638, Japan; ishidasu@med.hokudai.ac.jp; 2 Department of Ophthalmology, Nagoya City University Graduate School of Medical Sciences, Nagoya 467-8601, Japan; akikato@med.nagoya-cu.ac.jp; 3 Department of Ophthalmology, Nishida Hospital, Saiki 876-0047, Japan; kimotok@nisida-med.jp; 4 Department of Ophthalmology, Oita University Faculty of Medicine, Yufu 879-5593, Japan; mahoabe1104@oita-u.ac.jp; 5 Hikichi Eye Clinic, Sapporo 060-0807, Japan; thikichi@hikichi-eye.jp; 6 Department of Ophthalmology, Yayoi Hospital, Toyohashi 441-8106, Japan; wgoro@yayoi-hp.or.jp; 7 Takasaki Sato Eye Clinic, Takasaki 370-0036, Japan; tekutekuteku2000@gmail.com; 8 Matsuda Eye Clinic, Shiojiri 399-0738, Japan; matsu13eye@gmail.com; 9 Department of Ophthalmology, Kyushu University Hospital, Fukuoka 812-8582, Japan; asshyharyu@gmail.com; 10 Department of Ophthalmology, Mie University Graduate School of Medicine, Tsu 514-8507, Japan; hmatsu@med.mie-u.ac.jp

**Keywords:** brolucizumab, intraocular inflammation, triamcinolone acetonide, STTA, neovascular age-related macular degeneration, nAMD, retinal vasculitis, retinal vascular occlusion

## Abstract

**Highlights:**

**What are the main findings?**

- Under a treatment strategy incorporating sub-Tenon triamcinolone acetonide with intravitreal brolucizumab, the incidences of intraocular inflammation, retinal vasculitis, and retinal vascular occlusion were 2.5%, 1.25%, and 1.25%, respectively.
- Intraocular pressure elevation to ≥25 mmHg occurred in 12.5% of eyes; half of these eyes required topical treatment, and none required glaucoma surgery.

**What is the implication of the main finding?**

- These findings provide descriptive safety data for a treatment strategy incorporating sub-Tenon triamcinolone acetonide with intravitreal brolucizumab and may inform the design of future prospective comparative studies.

**Abstract:**

**Background/Objectives:** Brolucizumab is a potent therapeutic option for neovascular age-related macular degeneration (nAMD), but carries risks of intraocular inflammation (IOI), retinal vasculitis (RV), and retinal vascular occlusion (RO). This study evaluated the occurrence of these adverse events in eyes treated with intravitreal brolucizumab injection (IVBr) under a treatment strategy incorporating sub-Tenon injection of triamcinolone acetonide (STTA). **Methods:** This multicenter retrospective study included patients with nAMD who initiated IVBr treatment with STTA and were followed for at least 1 month. IVBr was administered under a treatment strategy incorporating STTA, with repeat STTA when more than 3 months had elapsed since the previous STTA. **Results:** The study included 80 eyes of 80 patients (59 men and 21 women; mean age, 74.5 ± 7.7 years) from 9 institutions. The mean number of previous intravitreal anti-vascular endothelial growth factor injections was 20.4 ± 18.3. The mean follow-up period was 6.8 ± 4.8 months. The mean numbers of IVBr and STTA administrations were 2.6 ± 1.6 and 1.7 ± 1.0 per eye, respectively. Three eyes (3.75%) developed adverse events: one developed IOI, one developed IOI with RV, and one developed RO without IOI or RV. **Conclusions:** IOI, RV, and RO occurred in 2.5%, 1.25%, and 1.25% of eyes, respectively. These data may help inform the design and sample-size planning of future prospective comparative studies.

## 1. Introduction

Neovascular age-related macular degeneration (nAMD) is a disease affecting middle-aged and older adults that causes central vision impairment [1]. Although treatment with anti-vascular endothelial growth factor (VEGF) agents has made it possible to maintain vision over the long term, cases resulting in severe visual impairment persist, and treatment can be challenging due to drug resistance [2]. Brolucizumab is used by intravitreal injection for nAMD [3] and diabetic macular edema (DME) [4] in many countries. It is a humanized single-chain anti-VEGF-A antibody fragment with a molecular mass of 26 kDa. This small size allows for an increase in the number of molecules that can be administered at a single injection; in fact, brolucizumab provides approximately 22-fold higher molar dosage than ranibizumab [5]. Therefore, brolucizumab has a strong impact on fluid control [3]. However, shortly after its approval in the United States in 2019, drug-related vision-threatening side effects were reported. Some patients developed intraocular inflammation (IOI) and retinal vasculitis (RV), and some cases were associated with retinal vascular occlusion (RO), resulting in severe visual impairment [6,7]. The pathogenesis of brolucizumab-associated inflammatory events remains to be elucidated. However, it is now recognized that these complications often respond well to steroids and can be controlled, and that early detection and intervention are crucial for preventing visual impairment [8,9,10,11]. Steroids are administered by eye drops, posterior sub-Tenon injections, intravitreal injections, and systemic administration, and treatments other than drops are often used for severe events in the posterior segment, such as RV or RO.

Several anti-VEGF agents are currently available for the treatment of nAMD, and switching to another agent is a common strategy when the response to a particular drug is insufficient. However, treatment response varies among individual patients, and previous studies have reported anatomical improvement after switching to brolucizumab in eyes with nAMD refractory to aflibercept or faricimab [12,13]. Therefore, brolucizumab may remain a clinically relevant therapeutic option in selected patients who do not achieve adequate disease control with other anti-VEGF agents. In such patients, strategies that may reduce the risk of brolucizumab-associated intraocular inflammatory adverse events could have clinical value.

Against this background, the possibility emerged that concomitant corticosteroid administration might reduce the occurrence of brolucizumab-associated intraocular inflammatory complications. Hikichi reported that in 30 cases in which a sub-Tenon injection of triamcinolone acetonide (STTA) was administered simultaneously with an intravitreal brolucizumab injection (IVBr), brolucizumab-associated intraocular complications (IOI, RV, and RO) did not occur [14]. Furthermore, reports of elevated aqueous humor flare values and inflammatory factors in cases of brolucizumab-associated IOI noted that the combined use of STTA with IVBr following the IOI prevented recurrence and did not increase flare values [15,16].

However, evidence regarding concomitant STTA with IVBr remains limited, particularly across different clinical settings, and the previously reported prophylactic series consisted of only 30 eyes from a single center [14]. Therefore, the present retrospective study evaluated a treatment strategy incorporating STTA with IVBr in a cohort of 80 eyes from nine centers to provide additional real-world information regarding the occurrence of brolucizumab-associated IOI, RV, and RO, as well as steroid-related adverse events.

## 2. Subjects and Methods

This study was a multicenter, retrospective study. The study included patients who received IVBr for nAMD under a treatment strategy incorporating STTA at participating institutions (Hokkaido University Hospital, Nagoya City University Hospital, Oita University Hospital, Hikichi Eye Clinic, Yayoi Hospital, Takasaki Sato Eye Clinic, Matsuda Eye Clinic, Kyushu University Hospital, Mie University Hospital) from 25 May 2020 to 31 March 2022. The 30 eyes included in the previous report by Hikichi [14] were treated through 31 August 2021 and were not included in the present study. At Hikichi Eye Clinic, only cases treated on or after 1 September 2021 were included in the present study; therefore, there was no overlap between the two cohorts. As shown in Supplementary Figure S1, inclusion criteria were eyes with at least 1-month follow-up after the initial IVBr + STTA. When both eyes were treated, only the eye that received treatment first was included. Exclusion criteria were eyes with previous brolucizumab-associated intraocular complications, and eyes with no follow-up examination within 2 months after the initial IVBr. Follow-up was stopped (i) when IVBr was administered alone more than 3 months after the last STTA, (ii) when another anti-VEGF drug was administered, (iii) when there was no follow-up visit for more than 2 months after IVBr, (iv) when IOI, RV, or RO developed, (v) when there was an interval of 4 months or more between follow-up visits, and (vi) when a macular hole developed. During the observation period, IVBr could be administered without additional STTA when ≤3 months had elapsed since the most recent STTA. If IVBr was required more than 3 months after the most recent STTA, additional STTA was administered. The 3-month interval was selected pragmatically on the basis of previous reports suggesting sustained ocular effects after periocular triamcinolone administration. In a clinical study of posterior juxtascleral triamcinolone acetonide, treatment effects were observed at 3 months and, for some outcomes, remained detectable at 6 months [17]. Previous pharmacokinetic work has also demonstrated vitreous penetration of triamcinolone after posterior sub-Tenon administration, although substantial interindividual variability was observed [18].

IVBr was administered at 6 mg/0.05 mL. STTA was performed using triamcinolone acetonide, which was used off-label for this purpose. The STTA dose was 40 mg/1 mL at Hokkaido University and 20 mg/0.5 mL at the other facilities. The injection site was selected at the discretion of the treating ophthalmologist and included the inferotemporal, inferonasal, superonasal, or superotemporal quadrant. The injection technique also varied among physicians: some administered triamcinolone acetonide into the sub-Tenon space using a blunt cannula after conjunctival incision, whereas others used a 27-gauge sharp needle without conjunctival incision. The order of IVBr and STTA administration was not standardized. In most cases, the two injections were performed during the same visit, with the second injection administered within 5 min of the first. In six treatment episodes involving five patients, IVBr was administered on a different day after STTA: the interval was 6 days in two episodes, 7 days in one, 10 days in one, 14 days in one, and 15 days in one.

IOI, RV, and RO were diagnosed by the treating ophthalmologists at each participating center based on clinical examination and available imaging findings. Because of the retrospective, multicenter nature of the study, no prospectively standardized diagnostic criteria or grading system for anterior chamber or vitreous inflammation was shared among the participating centers. No central adjudication or independent masked review of adverse events was performed. However, the clinical images of the adverse-event cases presented in the figures were reviewed by the coauthors to confirm that the imaging findings were consistent with the reported diagnoses. Follow-up examinations after IVBr were scheduled according to routine clinical practice at each participating center, and no uniform post-injection examination schedule was predefined. For the purposes of this study, the observation period was terminated if no follow-up examination was performed within 2 months after an IVBr injection.

Best-corrected visual acuity (BCVA) was measured in decimal scale values and converted to the logarithm of the minimum angle of resolution (logMAR) for statistical analysis. Intraocular pressure (IOP) was measured using either a non-contact tonometer or a rebound tonometer, depending on the participating center and the individual patient. IOP elevation was defined as an IOP of ≥25 mmHg during the observation period.

Continuous variables were summarized using means and standard deviations, medians and interquartile ranges (IQRs), and ranges, as appropriate; categorical variables were summarized using counts and percentages. Because only one eye per patient was included in the final analysis, eye-level and patient-level adverse-event proportions were equivalent. Exact 95% confidence intervals (CIs) for adverse-event proportions were calculated using the Clopper–Pearson exact binomial method. To account for differences in follow-up duration among study eyes, total observation time was calculated in eye-years, and incidence rates were expressed as events per 100 eye-years with exact Poisson 95% CIs. The observation period for each eye was calculated from the first IVBr administered concomitantly with STTA to the predefined end of observation. The duration of observation after each IVBr exposure was also summarized using the median and IQR. There were no missing data for the variables included in the analyses. The statistical analyses were exploratory and were not prespecified.

An exploratory sample size calculation was performed for a future prospective comparative study using a two-sample test for proportions, assuming a 6-month primary evaluation period. Assuming an incidence of 10% in the IVBr-alone group and incidences of either 2.5% or 5.0% in the IVBr treatment strategy incorporating STTA group, the required sample size was estimated with a two-sided significance level of 0.05, a statistical power of 80%, and a 1:1 allocation ratio. The sample size calculation was performed using the `power.prop.test` function in R software (R Foundation for Statistical Computing, Vienna, Austria).

All statistical analyses were performed using R software, version 4.0.3.

## 3. Results

### 3.1. Baseline Data

The study included 80 eyes of 80 patients (59 males and 21 females), with a mean age of 74.5 ± 7.7 years (Table 1). The mean number of previous intravitreal anti-VEGF injections (ranibizumab, aflibercept, or brolucizumab) was 20.4 ± 18.3, and only one eye was treatment-naïve. Four eyes had previously received IVBr monotherapy; 3 eyes had received 1 injection and 1 eye had received 5 injections. Twenty-seven eyes had previously received photodynamic therapy (PDT) with verteporfin, and one eye had undergone vitrectomy. Among the nAMD subtypes, polypoidal choroidal vasculopathy (PCV) accounted for 56%, type 1 macular neovascularization (MNV) for 40%, and type 3 MNV for 4%. Regarding lens status, 54 eyes were phakic and 26 eyes were pseudophakic. No eye had a baseline IOP > 20 mmHg.

### 3.2. Clinical Outcomes

The results of the follow-up period are summarized in Table 2. The mean follow-up period was 6.8 ± 4.8 months. Among the 80 study eyes, observation was continued until the end of the study period in 39 eyes (Supplementary Figure S1). In the remaining 41 eyes, observation was terminated because of subsequent IVBr without concomitant STTA ≥ 3 months after the last STTA in 17 eyes, switching to another anti-VEGF agent in 12 eyes, development of IOI, RV, or RO in 3 eyes, no follow-up visit for >2 months after IVBr in 7 eyes, interruption of follow-up for ≥4 months in 1 eye, and development of a macular hole in 1 eye. In the case of macular hole, vitreomacular traction syndrome was present from the beginning of the follow-up. One eye received concomitant PDT at the initial treatment. This eye was followed for 11 months, did not meet any of the predefined criteria for termination of observation, and developed no IOI, RV, or RO during the observation period. The mean number of IVBr was 2.6 ± 1.6 injections/eye, and the mean number of STTA was 1.7 ± 1.0 injections/eye. Seventy-five doses of IVBr alone were administered to 43 eyes. Mean logMAR BCVA was 0.47 ± 0.51 at baseline and 0.41 ± 0.43 at the final visit.

IOP elevation to ≥25 mmHg occurred in 10 eyes (12.5%) after 11 of the 134 STTA treatments (Table 2). Nine eyes developed elevated IOP within 1–4 months after STTA, whereas one eye developed elevated IOP 10 months after treatment. The mean IOP at the time of STTA administration was 16.1 ± 4.1 mmHg, and the mean maximum IOP among eyes with IOP elevation was 28.8 ± 4.9 mmHg. Five eyes (6.25%) were treated with anti-glaucoma eye drop(s) for 1 to 2 months, and no eyes required glaucoma surgery. IOP increased in 8 of 66 eyes (12.1%) who received 20 mg of STTA and in 2 of 14 eyes (14.3%) who received 40 mg of STTA. Among eyes with IOP elevation, the maximum IOP was a median of 25.5 mmHg (range, 25–32 mmHg) in the 20-mg group, whereas the maximum IOP values in the two eyes in the 40-mg group were 33 and 39 mmHg.

There were 54 phakic eyes, of which 2 (3.7%) underwent cataract surgery. These two eyes had received STTA two and three times, respectively.

### 3.3. Incidence and Clinical Characteristics of Brolucizumab-Associated Intraocular Complications

The total observation time for the 80 study eyes was 537 eye-months (44.75 eye-years). The median observation period per eye was 5 months (IQR, 3–9.25 months). A total of 208 IVBr injections were administered during the observation period. The median observation period after each IVBr exposure was 2 months (IQR, 1–3 months).

At the eye level, IOI, RV, and RO occurred in 2 of 80 eyes (2.50%; 95% CI, 0.30–8.74%), 1 of 80 eyes (1.25%; 95% CI, 0.03–6.77%), and 1 of 80 eyes (1.25%; 95% CI, 0.03–6.77%), respectively. At the injection level, three adverse-event episodes occurred during 208 IVBr administrations. Based on a total observation time of 44.75 eye-years, the incidence rates of IOI, RV, and RO were 4.47 (95% CI, 0.54–16.14), 2.23 (95% CI, 0.06–12.45), and 2.23 (95% CI, 0.06–12.45) events per 100 eye-years, respectively. No IOI, RV, or RO occurred in the five patients involved in the six treatment episodes in which STTA was administered 6–15 days before IVBr.

An exploratory sample size calculation was performed to estimate the number of eyes required for a future prospective comparative study of IVBr with and without concomitant STTA. The estimated sample sizes were 163 eyes per group (326 eyes in total) when incidences of 10% and 2.5% were assumed, and 435 eyes per group (870 eyes in total) when incidences of 10% and 5.0% were assumed, respectively.

**Figure 1:**
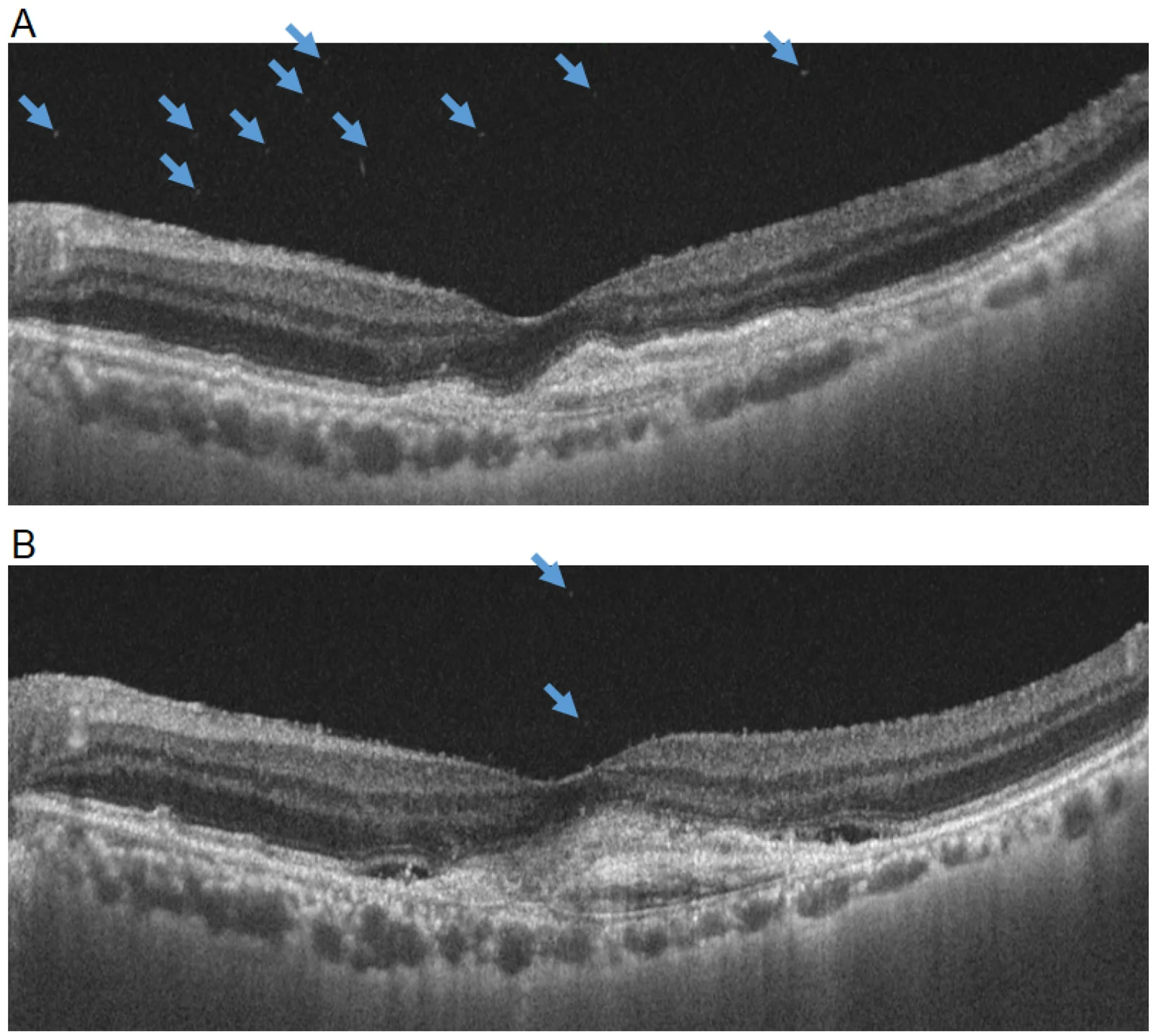
A 77-year-old man with intraocular inflammation. (**A**) Optical coherence tomography image of a horizontal section through the macula 47 days after the first intravitreal brolucizumab injection. Multiple hyperreflective dots (arrows) were observed in the vitreous cavity. (**B**) On day 96, the vitreous cells disappeared with no treatment.

The patient with IOI was a 77-year-old man with type 1 MNV who had received 7 aflibercept injections, 2 PDTs, and 2 STTAs. Two months after the last aflibercept injection, the patient was switched to IVBr with 40 mg-STTA (day 0). On day 15, he remained asymptomatic, and ophthalmic examination revealed no evidence of intraocular inflammation. Shortly thereafter, however, he noticed floaters. At the visit on day 47, anterior vitreous cells were observed, and optical coherence tomography demonstrated multiple hyperreflective dots in the vitreous cavity (Figure 1). No specific treatment was given, and on day 96, the vitreous cells had disappeared. His decimal BCVA was 0.5, unchanged from 0.6 at the start of the brolucizumab. This patient had neither diabetes nor hypertension.

**Figure 2:**
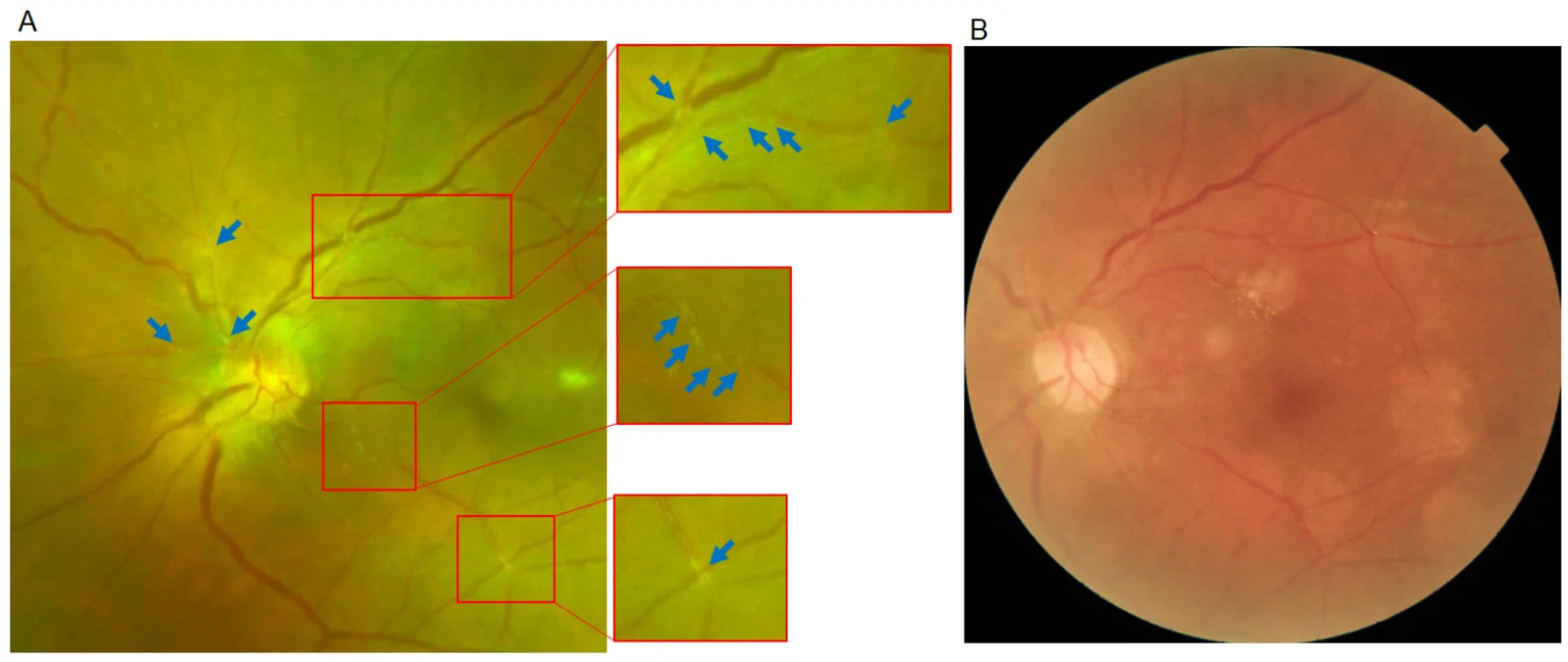
A 54-year-old woman with intraocular inflammation and retinal vasculitis (RV). (**A**) Fifteen days after the 2nd brolucizumab injection, segmental whitish vascular sheathing involving retinal arterioles and venules was observed (arrows), without retinal whitening or other funduscopic findings suggestive of retinal arterial occlusion. (**B**) Four months after the onset, the RV improved.

The patient with IOI and RV was a 54-year-old woman with PCV who had received 10 previous aflibercept injections, with the last one taken 1 month ago. The patient received IVBr with concomitant STTA 20 mg, and 2 months later received IVBr alone. At a routine visit 15 days after the second IVBr, occasional cells in the anterior chamber, a few keratic precipitates, and vitreous opacities were observed. Fundus examination showed segmental whitish vascular sheathing involving both arterioles and venules (Figure 2A). The vessels remained visible distal to these changes, and no retinal whitening or edema suggestive of retinal arterial occlusion was observed. The patient had no subjective symptoms. Based on these findings, RV was clinically diagnosed, although FA was not performed and the diagnosis was therefore not angiographically confirmed. STTA 20 mg was administered. One week later, the inflammatory and retinal vascular findings had not improved, and an additional 20 mg of STTA and betamethasone eye drops (4 times daily) were administered. Nine days later, the anterior chamber inflammation had resolved and the vitreous opacities had improved, whereas the retinal vascular findings remained unchanged. The patient was then treated with monthly aflibercept 3 times for increased subretinal fluid, and 4 months after the onset of IOI+RV, the RV findings improved (Figure 2B). Her decimal BCVA was 0.5 on the first IVBr, 0.7 on the second IVBr, then dropped to 0.5, and finally 0.4 after the switch to aflibercept. This patient had both diabetes and hypertension.

**Figure 3:**
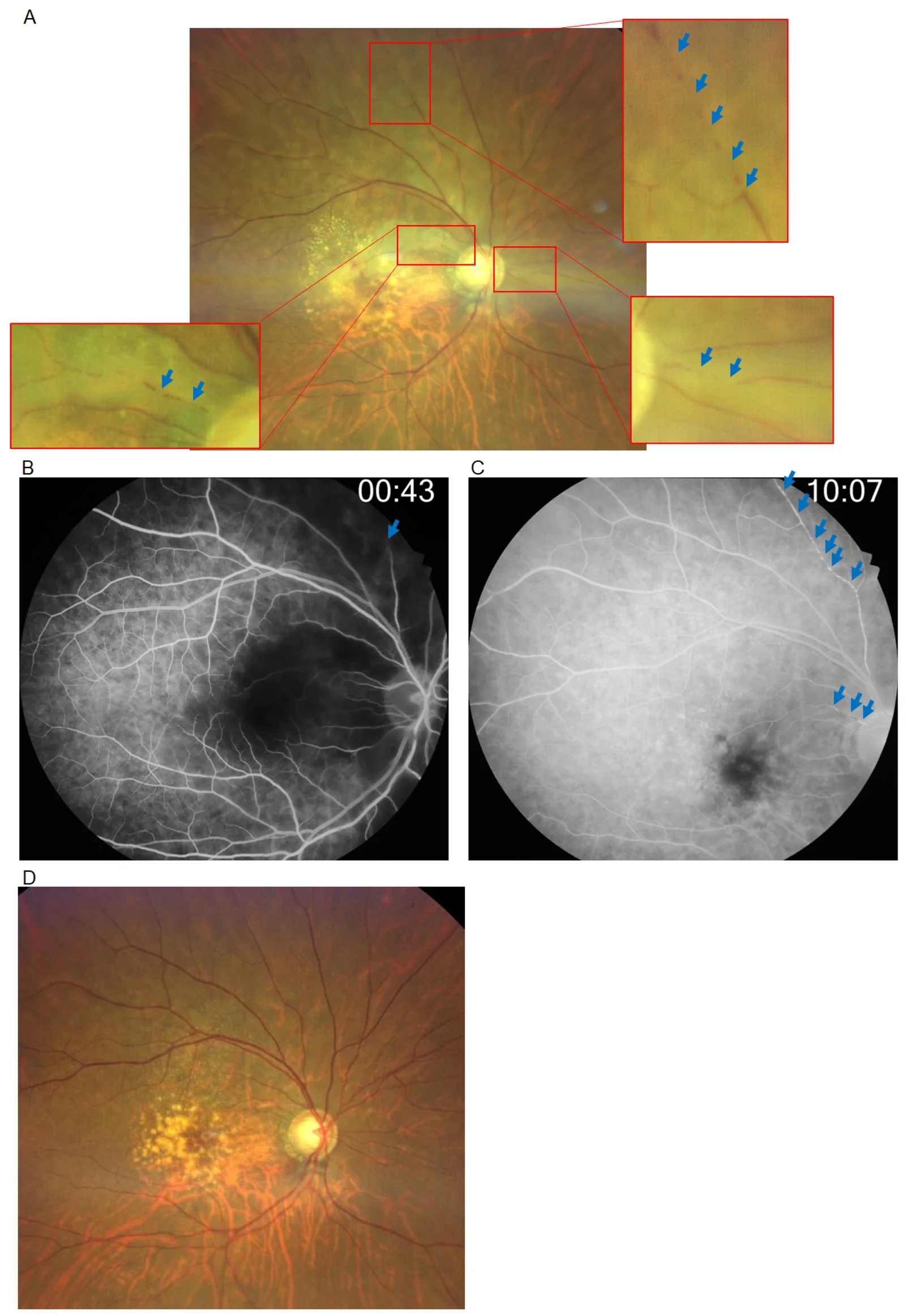
An 83-year-old woman with retinal vascular occlusion (RO). (**A**) Color fundus photography obtained 25 days after the first brolucizumab injection showed multiple ROs (arrows). Marked retinal edema was also present in these areas. (**B**) Early-phase fluorescein angiography demonstrated choroidal filling delay in the superior, temporal, and inferior regions of the optic disc, and an interruption of arterial blood flow in the superior retina (arrow). (**C**) Late-phase fluorescein angiography demonstrated a boxcarring appearance in multiple affected retinal arteries (arrows), consistent with markedly reduced retinal arterial blood flow, without angiographic evidence of retinal vasculitis. (**D**) One month after the onset, the RO lesions had improved.

The patient with RO was an 83-year-old woman with type 1 MNV who had received 3 previous ranibizumab injections, with the last one taken 2 months ago. On the morning of the 25th day after the first IVBr with simultaneous 20 mg-STTA, the patient experienced sudden visual obscuration, described as a dark cloud over her vision. There were no inflammatory findings in the anterior chamber or vitreous cavity, but her decimal BCVA deteriorated from 0.2 to 0.09, and fundus examination showed multifocal ROs involving at least three arteriolar branches, with retinal whitening along the affected areas (Figure 3A). FA demonstrated markedly delayed retinal arterial perfusion, with interruption of arterial filling in the early phase and a boxcarring appearance in multiple affected retinal arteries in the late phase, consistent with markedly reduced retinal arterial blood flow (Figure 3B,C) [19,20]. There was no angiographic evidence of retinal vasculitis. The patient was followed up with oral kallidinogenase only. One month after the onset, fundus findings improved (Figure 3D), but decimal BCVA worsened to 0.02 at 2 months. Fortunately, BCVA then slowly recovered, reaching 0.15 one year after the onset of RO. The patient’s medical history included aortic regurgitation, surgery for a gastric ulcer 50 years ago, surgery for gallstones 10 years ago, and surgery on the right knee one year ago. There was no history of diabetes or hypertension. Blood tests performed seven months after the onset of RO showed a low eGFR of 28.6 mL/min/1.73 m^2^, an antinuclear antibody titer of ≥1:40, and an elevated rheumatoid factor level of 150 IU/mL, whereas CRP was negative.

## 4. Discussion

### 4.1. Brolucizumab-Associated Intraocular Complications

In this study, we retrospectively investigated the incidence of the brolucizumab-associated intraocular complications among eyes treated with IVBr under a treatment strategy incorporating STTA in patients with nAMD. The results showed that the incidence of IOI, RV, and RO were 2.5%, 1.25%, and 1.25%, respectively. In the case of RO, multifocal retinal arterial occlusion was observed without accompanying signs of inflammation. Arterial occlusion unrelated to brolucizumab remains an important differential diagnosis. Although no diabetes or hypertension was documented, a comprehensive systemic evaluation for alternative embolic or vascular causes was not available, and such causes could not be excluded. Markedly reduced retinal arterial blood flow, including a boxcarring appearance on late-phase FA, was observed in multiple affected arterial branches. Although alternative systemic causes could not be excluded, the temporal relationship to brolucizumab administration made an association with brolucizumab clinically plausible.

Reported incidences of brolucizumab-associated intraocular complications in patients with nAMD are summarized in Table 3. The reported incidences varied among studies. Previous studies have identified female sex and Japanese ethnicity as risk factors for IOI [10,11,21], and higher incidences have also been reported in Japanese cohorts. Among studies of Japanese patients who received IVBr alone without concomitant STTA, the reported incidences of IOI, RV, and RO ranged from 9.4% to 15.6%, 2.6% to 11.1%, and 1.6% to 3.3%, respectively [21,22,23].

In the present study, which evaluated a treatment strategy incorporating periodic STTA with IVBr, the crude proportions of IOI, RV, and RO were 2.5% (95% CI, 0.30–8.74%), 1.25% (95% CI, 0.03–6.77%), and 1.25% (95% CI, 0.03–6.77%), respectively. Although the observed proportions of IOI and RV were numerically lower than those reported in previous studies of Japanese patients receiving IVBr alone, direct comparisons are limited by the absence of a concurrent control group and by substantial differences in baseline characteristics among the cohorts.

Women accounted for 26.3% of our cohort, compared with 14.2% in the study by Maruko et al. and 16.7% in the study by Mukai et al. [21,22]. In addition, our cohort was highly treatment-experienced, with a mean of 20.4 previous anti-VEGF injections, whereas 33.9% of patients in the study by Maruko et al. and 81% of eyes in the study by Mukai et al. were treatment-naïve, and the HAWK PCV subgroup consisted of treatment-naïve eyes [21,22,23]. The proportion of PCV also differed among studies; PCV accounted for 56% of our cohort, whereas the HAWK subgroup reported by Ogura et al. consisted exclusively of eyes with PCV [23]. These differences in baseline characteristics, together with differences in study design and follow-up, preclude direct comparison of crude adverse-event proportions across studies. Accordingly, the lower numerical incidence observed in the present study cannot be attributed to concomitant STTA administration on the basis of these historical comparisons, and the present findings do not establish whether concomitant STTA reduces the incidence of brolucizumab-associated adverse events.

Nevertheless, the observed event frequencies may provide useful information for planning future prospective comparative studies. In designing such a study, a 6-month primary evaluation period may be reasonable. Brolucizumab-associated inflammatory events have been reported to occur predominantly during the early treatment phase; in one prospective analysis, 81.1% of IOI-related events occurred after the first three brolucizumab injections [8]. In addition, a post hoc analysis of the HAWK and HARRIER trials showed that approximately 75% of IOI, RV, and RO-related events occurred within 6 months after the first brolucizumab injection [24]. For the exploratory sample size calculation, an incidence of 10% in the IVBr-alone group was assumed on the basis of previous Japanese studies, in which IOI incidences of approximately 9–16% were reported [21,22,23]. Incidences of 5.0% and 2.5% in the IVBr treatment strategy incorporating STTA group were selected as clinically meaningful target reductions, corresponding to relative reductions of 50% and 75%, respectively, from the assumed 10% incidence. Under these assumptions, 435 eyes per group would be required to detect a reduction from 10% to 5.0%, whereas 163 eyes per group would be required to detect a reduction from 10% to 2.5%, with 80% power and a two-sided significance level of 0.05. These estimates do not account for loss to follow-up; therefore, the sample size of an actual prospective study would need to be increased according to the anticipated dropout rate. A prospective comparative study with an appropriate control group would be required to determine whether a treatment strategy incorporating STTA has a preventive effect on brolucizumab-associated adverse events.

The availability of alternative anti-VEGF agents should be considered when the risk of brolucizumab-associated intraocular adverse events is a concern. Concomitant STTA should not be regarded as routine prophylaxis for all patients receiving brolucizumab. Rather, its potential role, if any, may be limited to selected patients in whom continued brolucizumab treatment is considered clinically beneficial after weighing the potential benefit of brolucizumab against both its inflammatory risks and the steroid-related risks of STTA. Because the present study lacked a control group, whether concomitant STTA reduces the risk of brolucizumab-associated adverse events remains uncertain.

### 4.2. Steroid-Associated Intraocular Complications

The main ocular safety concerns associated with STTA include cataract progression and IOP elevation. Bonini-Filho and colleagues reported no cases of cataract progression over a 24-week period in a randomized study of patients with DME (mean age, 61 years) who underwent STTA [30]. In the current study, 2 of 54 phakic eyes (3.7%) underwent cataract surgery during a mean follow-up period of 6.8 months. However, direct comparison with the previous study is limited by differences in patient characteristics, including age and underlying retinal disease. Moreover, because of the relatively short follow-up period, the present study cannot establish the long-term lenticular safety of concomitant STTA.

Previous studies indicated that with 20-mg STTA, 3.7% of patients developed an IOP of 24 mmHg or higher, 30% developed an IOP of 21 mmHg or higher, and with 40-mg STTA, 13.5% developed an IOP of 24 mmHg or higher, and 30.7% developed an IOP of 21 mmHg or higher [31,32,33]. Only about 1% required glaucoma surgery in these studies. In the current study, 12.5% of patients developed an IOP of 25 mmHg or higher, half of whom required anti-glaucoma eye drop treatment, and none required glaucoma surgery. Although steroid-induced ocular hypertension remains a concern, IOP was controlled with observation or topical treatment in all eyes that developed IOP elevation, and none required glaucoma surgery.

In this study, the follow-up period after STTA was as short as one to two months for some patients, suggesting that the incidence of these complications may be underestimated.

### 4.3. Limitations of the Current Study

This study has several limitations. First, it was retrospective and noncomparative, and the decision to use STTA was not standardized across participating centers. Some centers administered STTA routinely with brolucizumab, whereas others determined its use based on the treating physician’s judgment. Therefore, selection bias related to treatment selection cannot be excluded. In addition, eyes with a history of brolucizumab-associated complications were excluded, which may have preferentially excluded eyes at higher risk of recurrent adverse events. However, because re-administration of brolucizumab after such complications would not generally be considered in routine clinical practice, the study was intended to evaluate the occurrence of new brolucizumab-associated adverse events in eyes without a prior history of these complications. Accordingly, the present estimates should be interpreted as applying to this target population. Finally, the absence of a concurrent IVBr-alone control group precludes determination of whether a treatment strategy incorporating STTA reduces the risk of brolucizumab-associated adverse events.

Second, follow-up duration and visit intervals varied among participating centers and individual patients. Although incidence rates based on eye-time were additionally reported, mild or transient IOI events may have gone undetected in eyes with longer intervals between visits. Furthermore, observation ended before study completion in a substantial proportion of eyes for several clinically determined reasons, including subsequent IVBr without STTA, switching to another anti-VEGF agent, and insufficient follow-up. Therefore, informative censoring with respect to IOI, RV, and RO cannot be excluded. For steroid-related adverse events, particularly cataract progression, informative censoring may be of particular concern because STTA may have been discontinued in some eyes because of emerging steroid-related ocular changes.

Third, IOI, RV, and RO were diagnosed by the treating ophthalmologists based on routine clinical examination and available imaging findings, without prospectively standardized diagnostic or grading criteria, central adjudication, or a uniform imaging protocol. Therefore, diagnostic misclassification and intercenter variability cannot be excluded.

Fourth, the number of IOI and other adverse events was small, resulting in wide confidence intervals and limited precision of the estimated event frequencies. The small number of events also precluded a reliable assessment of potential predictive factors. In addition, because of the small number of IOP elevations and the confounding between STTA dose and treatment center, the present study does not allow conclusions regarding the comparative efficacy or safety of 20-mg versus 40-mg STTA. Given a previous case report showing that even 5 mg of STTA could prevent recurrence of IOI [34], further studies are warranted to determine the appropriate STTA dose.

Fifth, the duration of prophylactic anti-inflammatory activity of STTA against brolucizumab-associated inflammation has not been established. In one eye that developed IOI with RV, the event occurred approximately 2.5 months after the most recent STTA and approximately 2 weeks after the most recent IVBr (Supplementary Table S1). This observation suggests that any prophylactic effect of STTA, if present, may not be uniform throughout the assumed 3-month interval. Whether a shorter interval between STTA administrations could provide more consistent protection remains unknown and would need to be weighed against the risks associated with repeated corticosteroid administration.

Sixth, IOP was measured using either non-contact or rebound tonometry, with the measurement method varying among participating centers and individual patients. This heterogeneity may have introduced variability in IOP measurements and affected the classification of IOP elevation and, consequently, the estimated frequency of this adverse event.

Seventh, baseline glaucoma diagnosis, history of ocular hypertension, and use of IOP-lowering medications were not collected. Therefore, the potential influence of these baseline ocular factors on steroid-related IOP elevation could not be assessed.

Finally, the statistical analyses were exploratory and were not prespecified. Accordingly, the present estimates and exploratory sample-size calculations should be interpreted as hypothesis-generating and as information for the design of future prospective comparative studies rather than as confirmatory evidence.

## 5. Conclusions

This multicenter retrospective study provides real-world data on the frequency and spectrum of brolucizumab-associated and steroid-related ocular adverse events in 80 eyes treated with IVBr under a strategy incorporating periodic STTA. Because of the retrospective design and the absence of a concurrent control group, the present findings do not establish whether concomitant STTA reduces the risk of brolucizumab-associated adverse events. Nevertheless, these multicenter data, together with the exploratory sample-size estimates, may provide a useful basis for designing adequately powered prospective comparative studies to evaluate the potential preventive effect and safety of concomitant STTA.

## Figures and Tables

**Table 1:** Baseline characteristics of patients.

Male:female	59:21
Age (years)	74.5 ± 7.7 [54–87, median 75]
Number of past anti-VEGF injections	20.4 ± 18.3 [0–89, median 15]
Previous IVBr monotherapies (eyes)	4
Subtype of nAMD (eyes)	
PCV	45
Type 1 MNV	32
Type 3 MNV	3
Lens status; phakic:pseudophakic (eyes)	54:26
Treated with hypertension (patients)	38
Treated with diabetes (patients)	21

IVBr, intravitreal brolucizumab injection. MNV, macular neovascularization. nAMD, neovascular age-related macular degeneration. PCV, polypoidal choroidal vasculopathy. VEGF, vascular endothelial growth factor.

**Table 2:** Clinical outcomes of patients.

Follow-up period (months)	6.8 ± 4.8 [1–18, median 5]
Number of IVBr (injections/eye)	2.6 ± 1.6 [1–9, median 2]
Number of STTA (injections/eye)	1.7 ± 1.0 [1–6, median 1]
Number of IVBr monotherapy	75 injections, 43 eyes
BCVA (logMAR)	
Baseline	0.47 ± 0.51
Last visit	0.41 ± 0.43
Increase in IOP [≥25 mmHg] (eyes)	10 [12.5%]
Cataract surgery performed (eyes)	2 [3.7%]

BCVA, Best-corrected visual acuity. IOP, intraocular pressure. IVBr, intravitreal brolucizumab injection. logMAR, logarithm of the minimum angle of resolution. STTA, sub-Tenon injection of triamcinolone acetonide.

**Table 3:** Reported incidences of brolucizumab-related intraocular complications in studies with 30 eyes or more with nAMD.

Drug	Countries	Study Design	n	Follow-Up (Range)	IOI	RV	RO
IVBr w/o STTA	36 countries [24]	prospective	1088 ^#^	96 W	4.6%	3.3%	2.1%
IVBr w/o STTA	United States [25]	retrospective	10,654	97 D ^a^ (0–180)	2.4%	0.2%	0.4%
IVBr w/o STTA	United States [25]	retrospective	11,161	95 D ^a^ (0–180)	2.4%	0.2%	0.4%
IVBr w/o STTA	United States [26]	retrospective	172	42.5 D ^a^ from last IVBr	8.1%	0.6%	0.6%
IVBr w/o STTA	United States [27]	retrospective	51	NA	21.6%	2.0%	0%
IVBr w/o STTA	France [8]	prospective	210	48 W	11.4%	1.9%	1.9%
IVBr w/o STTA	France [8]	prospective	295	48 W	9.8%	2.0%	1.0%
IVBr w/o STTA	Germany and India [28]	retrospective	107	10.4 ± 2.0 M ^b^	0%	0%	1.0%
IVBr w/o STTA	Germany [29]	retrospective	63	16.04 ± 6.86 W ^b^ (2.86–27.43)	11.1%	1.6%	0%
IVBr w/o STTA	Japan [21]	retrospective	127	12.4 ± 4.7 W ^b^ (4–24)	9.4%	3.1%	1.6%
IVBr w/o STTA	Japan [22]	retrospective	90	3 M ^c^	15.6%	11.1%	3.3%
IVBr w/o STTA	Japan [23]	prospective	39	96 W	12.8%	2.6%	2.6%
IVBr with STTA	Japan [14]	retrospective	30	10.0 ± 0.6 M ^b^ (4–15)	0%	0%	0%
IVBr with STTA	Japan, Current study	retrospective	80	6.8 ± 4.8 M ^b^ (1–18)	2.5%	1.25%	1.25%

^#^ Of these, 358 patients received 3 mg of brolucizumab. ^a^ median. ^b^ average ± standard deviation. ^c^ no details given. D, days. IVBr, intravitreal brolucizumab injection. M, months. NA, not available. nAMD, Neovascular age-related macular degeneration. STTA, sub-Tenon injection of triamcinolone acetonide. W, weeks. w/o, without.

## Data Availability

The data presented in this study are available, but subject to restrictions, as the provision of data to third parties was not addressed in the opt-out statement. Please direct requests for the data to the corresponding author.

## Supplementary Materials

The following supporting information can be downloaded at https://www.mdpi.com/article/10.3390/jcm15218507/s1: Supplementary Figure S1. Flow diagram of study eyes. Eighty-five eyes with neovascular age-related macular degeneration (nAMD) treated with intravitreal brolucizumab injection (IVBr) concomitantly with sub-Tenon injection of triamcinolone acetonide (STTA) at nine participating centers and followed for at least 1 month were initially identified. Five eyes were excluded according to the predefined exclusion criteria, leaving 80 eyes for the final analysis. The reasons for exclusion and observation termination are shown. VEGF, vascular endothelial growth factor; IOI, intraocular inflammation; RV, retinal vasculitis; RO, retinal vascular occlusion. Supplementary Table S1. Clinical details of cases with brolucizumab-associated ocular complications.

## Abbreviations

The following abbreviations are used in this manuscript:

BCVA	best-corrected visual acuity
CI	confidence interval
DME	diabetic macular edema
IOI	intraocular inflammation
IOP	intraocular pressure
IVBr	intravitreal brolucizumab injection
logMAR	logarithm of the minimum angle of resolution
MNV	macular neovascularization
nAMD	neovascular age-related macular degeneration
PCV	polypoidal choroidal vasculopathy
PDT	photodynamic therapy
RO	retinal vascular occlusion
RV	retinal vasculitis
STTA	sub-Tenon injection of triamcinolone acetonide
VEGF	vascular endothelial growth factor
