# Supplementary material for: Brolucizumab-Associated Ocular Complications with Concomitant Sub-Tenon Triamcinolone Acetonide: A Multicenter Retrospective Study
Source: J Clin Med. 2026 Nov 2;15(21):8507. doi: 10.3390/jcm15218507 (PMC13764193; doi:10.3390/jcm15218507)
Supplement: Supplementary file 1 [file jcm-15-08507-s001.zip › jcm-4505732-supplementary.pdf]

Supplementary Materials:

Supplementary Figure S1. Flow diagram of study eyes. Eighty-five eyes with neovascular age-related macular degeneration (nAMD) treated with intravitreal brolucizumab injection (IVBr) concomitantly with sub-Tenon injection of triamcinolone acetonide (STTA) at nine participating centers and followed for at least 1 month were initially identified. Five eyes were excluded according to the predefined exclusion criteria, leaving 80 eyes for the final analysis. The reasons for exclusion and observation termination are shown.

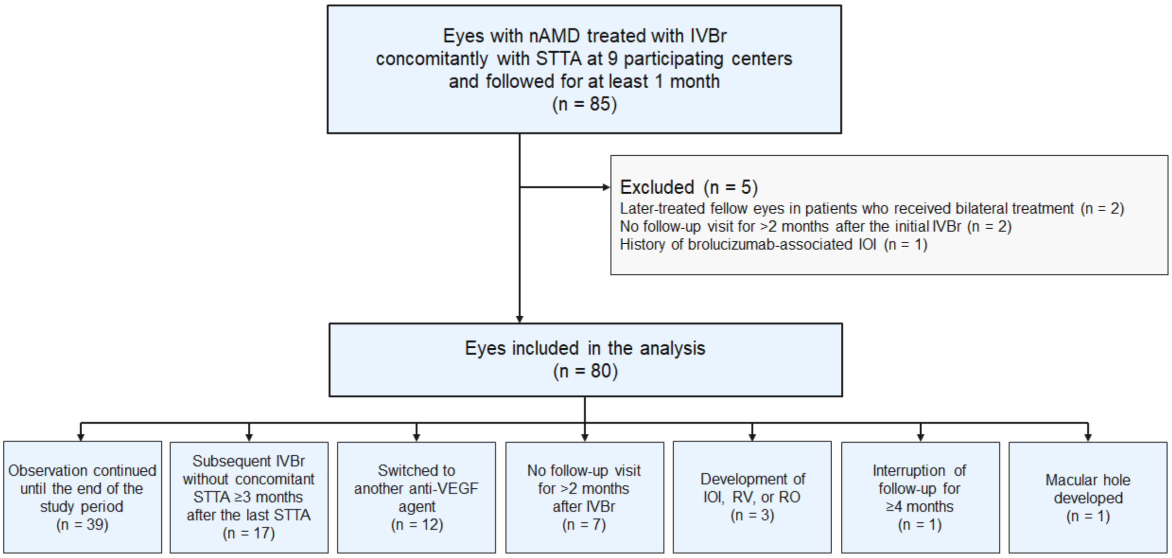

VEGF, vascular endothelial growth factor; IOI, intraocular inflammation; RV, retinal vasculitis; RO, retinal vascular occlusion.

Supplementary Table S1. Clinical details of cases with brolucizumab-associated ocular complications

	IOI	IOI+RV	RO
Age (years)	77	54	83
Gender	male	female	female
Time from most recent IVBr to subjective symptoms	>15 days	15 days*	25 days
Time from most recent STTA to subjective symptoms	>15 days	74 days*	25 days
Time from first IVBr+STTA to subjective symptoms	>15 days	74 days*	25 days

*As there were no subjective symptoms, the calculation was based on the date the adverse-event findings were confirmed.

IOI, intraocular inflammation; RV, retinal vasculitis; RO, retinal vascular occlusion;
IVBr, intravitreal brolucizumab injection; STTA, sub-Tenon injection of triamcinolone acetonide
